# COVID-19 and Myocarditis: Trends, Clinical Characteristics, and Future Directions

**DOI:** 10.3390/jcm14134560

**Published:** 2025-06-27

**Authors:** Mohammad Abumayyaleh, Tobias Schupp, Michael Behnes, Ibrahim El-Battrawy, Nazha Hamdani, Ibrahim Akin

**Affiliations:** 1 Department of Cardiology, Angiology, Haemostaseology and Medical Intensive Care, Medical Faculty Mannheim, University Medical Center Mannheim, Heidelberg University, 68167 Mannheim, Germany; tobias.schupp@umm.de (T.S.); michael.behnes@umm.de (M.B.); ibrahim.akin@umm.de (I.A.); 2 Department of Cardiology and Angiology, Bergmannsheil University Hospitals, Ruhr University of Bochum, 44789 Bochum, Germany; ibrahim.elbattrawy2006@gmail.com; 3 Institut für Forschung und Lehre (IFL), Department of Molecular and Experimental Cardiology, Ruhr-University Bochum, 44789 Bochum, Germany; nazha.hamdani@rub.de

**Keywords:** COVID-19, myocarditis, SARS-CoV-2, vaccination, heart failure

## Abstract

Summary: COVID-19, caused by SARS-CoV-2, has been associated with a range of cardiovascular complications, including myocarditis. This review aims to systematically present the clinical manifestations, underlying pathophysiological mechanisms, diagnostic approaches, and management strategies for both COVID-19-associated myocarditis and myocarditis related to SARS-CoV-2 vaccination. We conducted a literature search using the PubMed database, covering studies published up to early 2024. Search terms included combinations of “COVID-19”, “Coronavirus”, “SARS-CoV-2”, and/or “vaccination” with “cardiac injury”, “cardiac inflammation”, “myocarditis”. The reported prevalence of COVID-19-associated myocarditis varies between 2.3% and 5.0%, though myocardial injury is more frequently observed than confirmed myocarditis. Pathophysiological mechanisms include direct viral damage, immune-mediated injury, and molecular mimicry. Clinically, patients may present with chest pain, dyspnea, and fever. Diagnostic workup includes electrocardiography (ECG), troponin measurement, echocardiography, cardiac magnetic resonance imaging (cMRI), and in selected cases, endomyocardial biopsy (EMB). The management and disposition of COVID-19-associated myocarditis varies according to severity, especially to allow targeted treatment of complications. Glucocorticoids are a mainstay of treatment in severe cases. Myocarditis following SARS-CoV-2 vaccination is rare, more frequently reported in males under 30 years, and is generally associated with a favorable prognosis. Despite this, the benefits of vaccination continue to outweigh the risks. COVID-19 is associated with an increased risk of heart failure and other cardiovascular complications, underlining the importance of long-term follow-up and preventive strategies. Further research is needed to better understand the pathogenesis and optimal management of myocarditis in the context of COVID-19, with the goal of developing evidence-based therapeutic algorithms.

## 1. Introduction

The outbreak of coronavirus disease 2019 (COVID-19), which was caused by the novel severe acute respiratory syndrome coronavirus 2 (SARS-CoV-2), developed into a global health crisis [1]. In the early stages of infection, patients with SARS-CoV-2 were either asymptomatic or presented with severe pneumonia, organ dysfunction, and even death [2]. The risk factors for severe COVID-19 were determined, including older age, male sex, ethnicity, and the presence of underlying conditions such as cardiovascular diseases, hypertension, and chronic pulmonary diseases [3]. As a multi-organ disease, COVID-19 primarily affected the lungs, with respiratory failure being the most common complication [4]. However, cardiac complications, including acute cardiac injury, new-onset systolic dysfunction, pericardial effusion and, in a minority of cases, acute myocarditis, were also observed [4,5,6,7]. It has been reported that cardiac injury may be caused by direct cardiomyocyte infection with cardiomyocyte apoptosis [8,9]. This hypothesis was not corroborated by subsequent data [10,11]. A more compelling hypothesis suggests that cardiac injury may result from indirect mechanisms such as ischemia, fever, and hyperinflammation that are secondary to the existing cytokine storm [9]. Furthermore, additional mechanisms, including stress cardiomyopathy and acute right ventricular failure resulting from pulmonary embolism, have also been proposed [12,13].

Acute myocarditis is generally defined as an inflammatory process involving the myocardium, which may affect the cardiac conduction system and/or the pericardium. The global incidence of acute myocarditis is estimated at approximately four to fourteen cases per 100,000 individuals annually [14,15,16]. It can result from exposure to infectious agents, including coronaviruses, influenza, and parvovirus, as well as from systemic autoimmune diseases such as systemic lupus erythematosus, certain pharmacological agents (e.g., immune checkpoint inhibitors), and toxic substances, including chemotherapeutic drugs and vaccines [17,18,19,20]. In young individuals, acute myocarditis is recognized as a significant cause of sudden cardiac death (SCD) [21]. Clinically, patients may present with acute chest pain, dyspnea, palpitations, or, less commonly, syncope [22]. Diagnostic findings can include the presence of myocardial inflammatory infiltrates with nonischemic cardiomyocyte necrosis on endomyocardial biopsy (EMB) or autopsy [17]. Myocardial edema is frequently observed using cardiac magnetic resonance imaging (cMRI) [23,24]. However, according to the European Society of Cardiology’s (ESC) position statement, the diagnosis is considered clinically suspected myocarditis of uncertain etiology in the absence of EMB [25].

Nevertheless, COVID-19-associated myocarditis has been linked to various complications, including the development of dilated cardiomyopathy (DCM) [26]. De novo heart failure (HF) has been observed in up to one-quarter of hospitalized patients and in approximately one-third of those requiring intensive care unit (ICU) admission [13]. In such cases, the management of HF resulting from DCM should follow the same principles as those applied in idiopathic DCM [27]. Hospitalized patients with COVID-19-associated acute myocarditis, often accompanied by respiratory insufficiency, have received a range of antiviral or immunosuppressive treatments, including hydroxychloroquine, tocilizumab, remdesivir, lopinavir/ritonavir, interferon beta, colchicine, intravenous steroids, and intravenous immunoglobulins [28,29,30,31,32,33]. Furthermore, myocarditis induced by SARS-CoV-2 vaccination has been recognized as a rare cause of hospitalization during the COVID-19 pandemic [34,35]. However, a more frequently encountered condition is myocardial injury, defined by elevated troponin levels exceeding the 99th percentile upper reference limit [36].

The purpose of this review is to provide a comprehensive and systematic overview of the current evidence on COVID-19-associated acute myocarditis. In this context, we will describe the current and future challenges in treating acute myocarditis, as well as the potential risk of an increased incidence of HF.

## 2. Materials and Methods

We conducted a structured literature review using the PubMed database to identify relevant studies on COVID-19-associated myocarditis and myocarditis related to SARS-CoV-2 vaccination. The search included articles published up to early 2024. For COVID-19-associated myocarditis, search terms included combinations of “COVID-19”, “SARS-CoV-2”, and “Coronavirus” with keywords such as “myocarditis”, “cardiac injury”, “cardiac inflammation”, “cardiac involvement”, and “pericarditis”. For vaccine-associated myocarditis, search terms included combinations of “vaccination”, “mRNA vaccine”, and “immunization” with keywords such as “myocarditis”, “cardiac injury”, “cardiac inflammation”, and “cardiac involvement”. In addition to the database search, we screened the reference lists of selected articles to identify further relevant studies.

We included peer-reviewed original research articles, observational studies, clinical trials, case reports, case series, and systematic reviews. Only articles published in English were considered. Preprints, commentaries, and conference abstracts were excluded. Articles focusing on non-cardiac complications of COVID-19 or vaccination were also excluded unless they provided significant discussion of myocarditis.

Titles and abstracts were screened to determine initial eligibility, followed by full-text review for articles deemed potentially relevant. From the selected studies, we extracted data on population characteristics, clinical manifestations, diagnostic findings, treatment approaches, and reported outcomes.

## 3. Epidemiology

The reported prevalence of COVID-19-associated acute myocarditis varies depending on the specific criteria employed. It is important to differentiate this condition from cardiac injury resulting from multiple organ failure, hypoxia, and activation of the hemostatic system [37]. A total of 1597 athletes across 13 centers with confirmed COVID-19 were evaluated using cMRI. Among them, 2.3% exhibited either clinical or subclinical acute myocarditis, while only 0.31% presented with cardiac symptoms, reflecting the detected prevalence [38]. In a retrospective cohort study using electronic medical records from 718,365 hospitalized patients with COVID-19, 35,820 (5.0%) developed acute myocarditis [39]. Among hospitalized patients at 23 centers in the United States (US) and Europe, the prevalence of COVID-19-associated acute myocarditis was 2.4 (confirmed/probable) and 4.1 (possible) cases per 1000 COVID-19 hospital admissions [40]. However, these cases were identified based on clinical presentation and included only patients who presented with symptoms at the time of their hospital admission. The prevalence did not include asymptomatic patients, and large-scale data on asymptomatic myocardial involvement are lacking. In a multicenter autopsy study, lymphocytic myocarditis was identified in 3 of 21 consecutive patients diagnosed with COVID-19. It should be noted that the definition of myocarditis included the presence of multiple foci of inflammation associated with myocyte injury. Immunohistochemistry was used to analyze the composition of inflammatory cells [41]. Among 277 patients in autopsy studies, the prevalence of myocarditis was less than 2% [42]. However, acute myocardial injury was more prevalent than myocarditis in hospitalized patients with COVID-19. The incidence of myocardial injury ranged from 19.7% to 28.7% in different studies [43,44,45]. Table 1 presents the prevalence of COVID-19-associated acute myocarditis.

## 4. Pathophysiology

SARS-CoV-2 is a large, enveloped RNA virus that shares significant nucleotide sequence homology with other coronaviruses, including SARS-CoV and Middle East respiratory syndrome coronavirus (MERS-CoV) [46]. The virus utilizes ACE2 for cellular entry [47]. Transmembrane serine protease-2 (TMPRSS2), located on the plasma membrane, cleaves the viral spike protein, facilitating entry into the cytosol. Additionally, intracellular proteases such as the cathepsins in the endosomes contribute to this process [48]. ACE2 is predominantly expressed on cardiomyocytes, whereas TMPRSS2 is mainly found on endothelial cells, potentially enabling direct cardiac infection [49,50]. Cardiomyocytes can also express additional entry-related proteins, such as neuropilin-1 receptor (NRP1), integrin α5ß1, and cathepsins. These proteins may act as antigen-presenting cells (APCs), activating other immune cells, including dendritic cells and macrophages, and initiating specific T-helper cell responses [8,51,52,53]. The interaction between viral antigens and the resulting immune response can lead to cardiac injury, manifesting as myocarditis. In both COVID-19-related and autoimmune models of myocardial injury, myocardial infiltrates often consist of up to 80% macrophages, with fewer T and B lymphocytes, indicating a predominance of innate over adaptive immune response. Thus, referring to this process as “lymphocytic” myocarditis may be somewhat misleading. An additional potential mechanism of autoimmunity is molecular mimicry, wherein structural similarities between SARS-CoV-2 proteins and human peptides lead to cross-reactive immune responses. This mechanism may contribute to autoimmune-mediated myocardial damage, even in the absence of high viral load or direct viral invasion [54].

**Figure 1:**
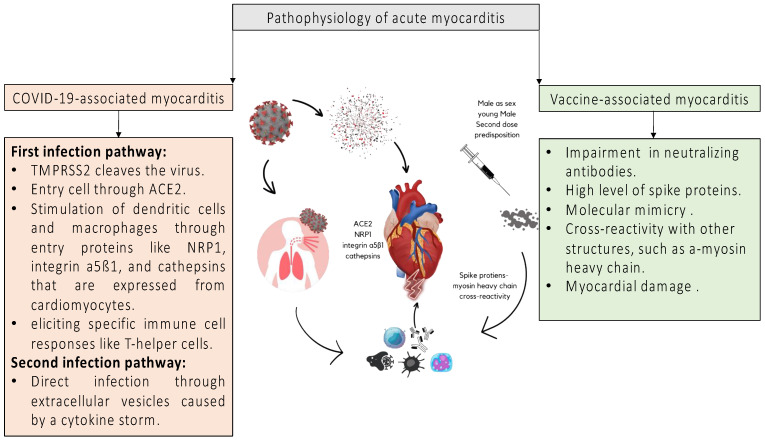
Pathophysiology of acute myocarditis in COVID-19-associated and its vaccine-associated acute myocarditis.

Two potential infection pathways in myocardial tissue are currently under investigation: direct infection and indirect injury via extracellular vesicles during a cytokine storm. Direct infection of cardiomyocytes is a plausible mechanism of myocarditis development [55]. Alternatively, the intense cytokine release associated with SARS-CoV-2 infection may also contribute. However, existing animal models have not shown that pro-inflammatory cytokines alone are sufficient to induce myocarditis [56]. Moreover, a high viral load does not appear necessary to trigger cardiac inflammation [57]. Figure 1 represents the proposed pathophysiological mechanisms of acute myocarditis.

## 5. Clinical Presentation and Diagnosis of COVID-19-Associated Acute Myocarditis

Patients with possible COVID-19-associated acute myocarditis may present with a range of symptoms, including those that are specific to the condition (e.g., chest pain, syncope, and palpitations) and those that are non-specific (e.g., respiratory symptoms such as dyspnea, cough, fever, and myalgias, or gastrointestinal symptoms like nausea and diarrhea) [40,58,59]. In this regard, dyspnea and cough were observed more in hospitalized patients with concomitant acute myocarditis and clinically diagnosed pneumonia than in those without pneumonia, respectively (78.3% vs. 35.5%) (69.6% vs. 29%) [40]. In previous studies on perimyocarditis, chest pain was the most frequently reported symptom, occurring in up to 95% of cases, followed by dyspnea (19–49%) and syncope in approximately 6% of cases [60,61,62]. Notably, fever was present in around 65% of patients [61]. An asymptomatic course was also reported in 5% of athletes with COVID-19-related cardiac involvement [63]. Additionally, flu-like symptoms may precede the onset of myocarditis. In this context, a large analysis with one-year follow-up showed a 7.9% risk of developing myocarditis following a respiratory tract infection with COVID-19 [64]. Other clinical presentations, including ventricular tachyarrhythmias, pericardial effusion, cardiogenic shock, and SCD, have also been described [29,31,65]. Cases of myositis and rhabdomyolysis have also been reported in patients with severe COVID-19 [66].

The electrocardiogram (ECG) has become an essential diagnostic tool in the emergency department. In general, our data showed that ECG abnormalities were present in hospitalized patients with COVID-19, including atrial fibrillation in approximately 7% of patients without diagnosed acute myocarditis, and prolonged QTc intervals as well as life-threatening arrhythmias with an incidence of 3.6% [67,68]. Nevertheless, ST-segment elevation was observed in patients with COVID-19 and perimyocarditis [65]. Based on our published and other available data, ECG remains an important diagnostic tool for the detection of arrhythmias or ST-segment deviations in patients with COVID-19-associated acute myocarditis. Patients exhibiting such ECG alterations should be classified as a high-risk group and subjected to monitoring due to the potential for malignant arrhythmias.

In terms of diagnostic utility, troponin testing is capable of detecting myocardial injury in the presence of necrosis [69,70]. It is important to note that myocarditis is not necessarily accompanied by necrosis. Consequently, the absence of elevated troponin does not necessarily rule out myocarditis but is more indicative of a severe form of myocarditis [71,72]. Troponin alone is an inadequate diagnostic tool for differentiating between myocardial injury and acute myocarditis, as it is typically elevated in both conditions. However, if troponin levels remain elevated after exclusion of myocardial ischemia for a patient with matching clinical symptoms for myocarditis, a diagnosis of myocarditis is highly probable and should be addressed by further diagnostic work-up [73]. Table 2 presents clinical presentation, ECG, troponin, and radiographic findings in COVID-19-associated acute myocarditis.

Transthoracic echocardiography can identify probable findings in patients with acute myocarditis, including reduced left ventricular ejection fraction (LVEF), segmental wall motion abnormalities, left ventricular thickening, and the presence of pericardial effusion when the pericardium is involved [59,81]. In patients with acute myocarditis, possible findings may include inferior and inferolateral wall hypokinesia, diastolic dysfunction, mild dysfunction in the right ventricle, and abnormal myocardial echogenicity [82]. LVEF serves as a prognostic indicator [61,82].

In patients with clinical suspicion of COVID-19-associated acute myocarditis, cMRI may be considered for diagnosis based on Lake-Louise criteria [83,84]. In this context, myocardial wall edema and T2 late gadolinium enhancement may occur in the setting of acute inflammation, whereas fibrotic remodeling may be more common in the later course of the disease [74]. Other potential causes can be excluded [24]. In addition, cMRI can be employed to monitor long-term outcomes or to monitor developing persistent inflammation [85,86]. However, the European Society of Cardiology Working Group on Myocardial and Pericardial Diseases has stated that cMRI should not replace EMB in diagnosing myocarditis and also should not delay EMB in life-threatening cases [25]. EMB should be acknowledged as the gold standard for confirming the diagnosis of acute myocarditis based on the Dallas criteria [25,87]. However, in clinical practice, EMB is not routinely performed, and as a result, the diagnosis is typically based on clinical suspicion. In many cases, the underlying etiology remains unclear, and the condition is therefore referred to as clinically suspected myocarditis of unknown origin. Regarding that, cMRI is the most sensitive non-invasive diagnostic tool for detecting myocardial and pericardial involvement, and it should be considered in clinically stable patients with suspected myocarditis. Some case reports of COVID-19-associated acute myocarditis demonstrated the presence of lymphocytic infiltrates, myocardial edema, and necrosis, while others did not [78,88]. Given that the complication rate tends to decline with increasing expertise, it would be preferable for experienced centers to perform EMB [89,90].

The standard EMB, particularly the LV approach, is crucial for definitively diagnosing myocarditis and obtaining information on its pathophysiology, particularly when the right ventricle is structurally and functionally normal [91,92]. Furthermore, the sensitivity of the procedure can be enhanced by collecting more specimens than the minimum recommended number (from 4 to 6 specimens) [25]. Qualitative criteria to enhance the diagnostic yield of EMB in acute myocarditis include the Marburg criteria, which were delineated in a position statement drafted by the ESC experts [25]. However, EMB should be considered in patients with severe HF or cardiogenic shock suffering from fulminant myocarditis, ventricular arrhythmias, high-degree atrioventricular block or chronic inflammatory cardiomyopathy [17,93]. The necessity for an EMB may arise during either the acute or chronic phase of myocarditis, depending on the urgency of the matter. Furthermore, angiography may be considered to rule out myocardial infarction, especially in patients with acute myocardial injury [94].

Nevertheless, despite these findings in non-invasive diagnostic work-up, no single finding is pathognomonic for COVID-19 associated acute myocarditis. Rather, these finding should be interpreted in context with symptoms and patient history. Table 2 shows the prevalence of clinical presentations, ECG findings, laboratory parameters, radiographic and echocardiographic findings, cMRI, and EMB in patients with COVID-19-associated acute myocarditis.

## 6. Management of COVID-19-Associated Acute Myocarditis

To date, there are no evidence-based guidelines for the management of COVID-19-associated acute myocarditis. In terms of mortality risk, patients are typically categorized into three groups. In general, patients with clinically suspected myocarditis should avoid intense physical activity. Expert consensus recommends refraining from intense sports for a period of 3 to 6 months. For athletes, a repeat risk assessment is advised before returning to sports to evaluate their functional status and ensure safe reintegration [95]. Specific recommendations regarding driving restrictions and recovery timeline remain undefined and should be individualized. These decisions depend on the patient’s underlying comorbidities and the presence of complications, such as myocarditis-related LV dysfunction or arrhythmias causing syncope.

Low-risk patients with normal findings in echocardiography and mild symptoms and without arrhythmias were observed as outpatients [74]. Depending on the presence of symptoms, LVEF, and the occurrence of arrhythmias, patients may be classified intermediate- or high-risk [17]. However, both groups should be admitted to an advanced center as inpatients and monitored as needed. Furthermore, a number of factors influence the decision to hospitalize patients. If there are predictors of an unfavorable prognosis, such as fever, a subacute course, and evidence of a large pericardial effusion, it is recommended that the patient be admitted to the hospital [96]. If the desired outcomes of anti-inflammatory treatment do not occur within one week, hospitalization may need to be considered as the next step [97]. During the monitoring period, there is no evidence-based recommendation. Beta-blockers may be utilized for the prevention of arrhythmia. In the event that LVEF is impaired, ACE inhibitors or ARBs should be initiated according to the ESC HF guidelines [27]. In cases of severely impaired LVEF, guideline-based therapy for HF should be initiated in stable patients [24]. If patients exhibit ventricular arrhythmias, it may be advisable to provide them with a wearable cardioverter defibrillator (WCD) until a decision can be made regarding defibrillator implantation [98,99].

Patients with rapid disease progression should be managed at advanced centers with intensive care and mechanical circulatory support (MCS). If pneumonia is present, consider administering treatment with steroids [100]. For patients with pericardial involvement, it is recommended to consider the use of non-steroidal anti-inflammatory drugs (NSAIDs) and colchicine therapy. In patients with hemodynamically relevant myocarditis or multisystem inflammatory syndrome in adults (MIS-A), intravenous steroids may be utilized. In addition, steroids may be employed in patients exhibiting biopsy-proven severe infiltrative changes or those presenting with fulminant myocarditis. Patients with HF should receive guideline-based therapy with follow-up [24]. In patients with suspected severe COVID-19-associated myocarditis, the use of intravenous corticosteroids may be considered [24,101]. In cases of recurrent or chronic inflammatory cardiomyopathy associated with autoimmune disorders, treatment should follow established guidelines for the underlying systemic condition [17]. Corticosteroids play a central role, often in combination with other immunosuppressive agents. Plasmapheresis may be employed in selected acute cases, such as myocarditis associated with antiphospholipid syndrome. In patients with refractory heart failure or cardiogenic shock, MCS, including left ventricular assist devices, or urgent heart transplantation may be necessary [17].

Myocardial injury in the context of COVID-19 has also been widely documented. This broad variability reflects the heterogeneous nature of its underlying causes, which may include acute coronary syndromes (type 1 myocardial infarction), demand ischemia (type 2 myocardial infarction), Takotsubo cardiomyopathy, sepsis, acute cor pulmonale due to macro- or microvascular pulmonary embolism, as well as injury related to chronic cardiovascular conditions such as preexisting HF. In some cases, acute viral infection may also unmask previously undiagnosed or subclinical heart disease. Given that multiple mechanisms may coexist in a single patient, determining the exact etiology of myocardial injury can be challenging, and establishing a targeted treatment strategy depends on accurate identification of the underlying cause [24]. In this review, due to the wide spectrum of myocardial injury mechanisms, we specifically focus on the management of myocarditis associated with SARS-CoV-2 infection and its vaccination.

## 7. Myocarditis After SARS-CoV-2-Vaccination; Should Vaccination Be Omitted?

Myocarditis following vaccination has been previously reported in the literature prior to the advent of SARS-CoV-2-vaccination [102]. However, vaccine-associated myocarditis in the context of SARS-CoV-2 vaccines has been relatively rare. Between December 2020 and June 2021, the United States Vaccine Adverse Event Reporting System (VAERS) reported 1226 possible cases of myocarditis after the administration of approximately 300 million mRNA vaccine doses. The majority of patients were male and under the age of 30 years old [103]. In a retrospective study conducted in Israel, it was observed that 5.1 million individuals who had received the full Pfizer-BioNTech mRNA vaccination, detecting 142 cases of acute myocarditis associated with SARS-CoV-2 vaccination [104]. However, individuals who had recovered from COVID-19 exhibited a fourfold higher risk of developing myocarditis compared to those who received SARS-CoV-2 vaccination [105]. As there is no systematic data regarding asymptomatic myocardial involvement after vaccination, it is possible that the data may be underestimated. Table 3 presents the incidence of vaccine-associated acute myocarditis.

**Table 3:** Incidence of vaccine-associated acute myocarditis.

		Vaccine-Associated Acute Myocarditis		
Country	Vaccine	Time Period	Incidence	References
US	BioNTech and Moderna	December 2020–June 2021	1226/300 million doses	[103]
Israel	BioNTech	December 2020–May 2021	117/10.2 million doses	[104]
Denmark	BioNTech and Moderna	October 2020–October 2021	269/4.1 million doses	[106]
US and Canada	BioNTech and Moderna	February–May 2021	20/3.5 million doses	[107]

**Table 4:** Clinical presentation, ECG, troponin, and radiographic findings in vaccine-associated acute myocarditis.

Clinical Presentation	Vaccine	Prevalence	References
Chest pain	Pfizer-BioNTech/Moderna–P/M/J/AZD/C *	90.1–100%	[108,109,110]
Syncope	P/M/J/AZD/C *	0.3%	[110]
Palpitations	P/M/J/AZD/C *	6.1%	[110]
Dyspnea	Pfizer-BioNTech/Moderna–P/M/J/AZD/C *	25–25.7%	[109,110]
Cough	P/M/J/AZD/C *	0.4%	[110]
Fever	Pfizer-BioNTech/Moderna–P/M/J/AZD/C *	11.9–32%	[109,110]
Myalgia	Pfizer-BioNTech/Moderna	6%	[109]
Gastrointestinal symptoms	Pfizer-BioNTech/Moderna–P/M/J/AZD/C *	5.7–11%	[109,110]
**ECG**			
ST-segment elevation	Pfizer-BioNTech/Moderna–P/M/J/AZD/C *	34.9–67.3%	[109,110]
Other abnormal ST changes	Pfizer-BioNTech/Moderna	9.6%	[109]
**Laboratory parameters**			
Troponin	Pfizer-BioNTech/Moderna–P/M/J/AZD/C *	97.6–98.1%	[109,110]
NT-proBNP	Pfizer-BioNTech/Moderna	69.6%	[109]
**Echocardiographic findings**			
LV dysfunction	Pfizer-BioNTech/Moderna–P/M/J/AZD/C *	23.2–44%	[109,110]
Wall motion abnormalities	Pfizer-BioNTech/Moderna	34%	[109]
Pericardial effusion	Pfizer-BioNTech/Moderna P/M/J/AZD/C *	1.8–22%	[109,110]
**cMRI**			
T2 elevation	Pfizer-BioNTech/Moderna P/M/J/AZD/C *	61.9–63.3%	[109,110]
Late gadolinium enhancement	Pfizer-BioNTech/Moderna P/M/J/AZD/C *	80.7–95.2%	[109,110]
Pericardial effusion	P/M/J/AZD/C *	4.2%	[110]

Behers et al. [109]: median of age 24 years (14–80 years old), time to symptom onset 3 days (1–90 days), vaccine: Pfizer 69.8% (first dose 13.2%, second dose 54.7%), Moderna 30.2% (first dose 5.7%, second dose 24.5%). Goyal et al. [110]: mean of age 25.5 ± 14.2 years old, time to symptom onset 4.01 ± 6.99 days, vaccine: mRNA-based vaccine (99.4%). *, Pfizer-BioNTech 73%, Moderna 26.4%, Janssen (J&J) 0.3%, AstraZeneca 0.2%, COVAXIN (Bharat Biotech) 0.04%, first dose 16.8%, second dose 83.2%. Abbreviation: ECG, electrocardiogram; ST-segment, ST segment is the flat, isoelectric section of the ECG between the end of the S wave (the J point) and the beginning of the T wave; NT-proBNP, N-terminal pro-brain natriuretic peptide; LV, left ventricular; cMRI, cardiac magnet resonance imaging.

The pathophysiology of COVID-19 vaccine-associated myocarditis is not understood at this time, but ongoing research may provide more information in the future. However, several hypotheses have been suggested. In the presence of predisposing factors such as age and male sex, the mRNA molecules themselves may be immunogenic, activating the innate immune system and leading to their destruction before they enter the host cells [111]. Yonker et al. reported that spike proteins may play a potentially important role in the development of COVID-19 vaccine-associated myocarditis [112]. In this context, a potential decrease or impairment in neutralizing antibodies has been discussed, resulting in higher levels of circulating spike proteins, with particular emphasis on the crucial role of anti-idiotype antibodies [113]. However, the mRNA molecules can also induce a systemic response resulting in myocardial damage. In this case, there should be evidence of involvement of other organs. Molecular mimicry between the spike proteins of SARS-CoV-2 and host self-antigens also seems theoretically possible [114]. Antibodies released in response to spike proteins may lead to cross-reactivity with other structures, such as a-myosin heavy chain, and result in myocardial damage as they attack it [115]. In addition, an increased dose of mRNA vaccine has been associated with the risk of myocarditis in groups at risk for vaccine-associated myocarditis [116,117]. Figure 1 represents the pathophysiology of vaccine-associated acute myocarditis. Regarding symptomatology, symptoms typically begin within the first few days after administration of the second dose of SARS-CoV-2 vaccine [118]. Almost all patients presenting with SARS-CoV-2-associated myocarditis present with chest pain (90.1–100%). This may reflect a selection bias to identify only symptomatic cases [108,109,110]. Troponin levels are elevated in most patients, peaking between 48 and 72 h after symptom onset [34]. In patients with suspected vaccine-associated myocarditis, cMRI is an invaluable diagnostic tool for establishing a diagnosis [119,120]. Table 4 illustrates the clinical presentation, ECG findings, troponin levels and radiographic findings of vaccine-associated acute myocarditis.

In accordance with current ESC guidelines, large population studies have demonstrated the safety of the SARS-CoV-2 vaccine, and anecdotal reports of vaccine-associated complications in patients with cardiomyopathy are available. It is recommended that all patients with cardiomyopathy receive the vaccine, especially those with signs or symptoms of HF, given the potential for worse outcomes in patients with cardiomyopathy who contract COVID-19 [121]. In general, patients exhibit a favorable prognosis, and the SARS-CoV-2 vaccine has a favorable benefit-to-risk ratio across all age and gender groups [30]. Data on evidence-based therapy is currently lacking. Nevertheless, some studies have utilized therapy with NSAIDs [122].

## 8. Outcomes and Long-Term Cardiac Complications Following COVID-19-Associated Myocarditis

An increased risk of cardiac arrhythmias and myocarditis was observed in COVID-19 patients compared to patients without COVID-19 with lower respiratory tract illnesses in 2019 [123]. Furthermore, in-hospital mortality rate for patients with myocarditis and concomitant SARS-CoV-2 infection was sixfold higher than that observed in patients with myocarditis alone [124]. Moreover, fatal myocarditis cases attributed to the SARS-CoV-2 vaccine have been documented [125]. The mortality rate in these groups remains high [126]. Nevertheless, the quality of the current evidence on the causal relationship and incidence of COVID-19 vaccine-associated myocarditis and HF remains low [127]. A cohort of 587,330 hospitalized patients in the US was divided into two groups; half had COVID-19, and the other half did not. During the 467-day follow-up period, HF developed in 10,979 previously unaffected patients. After adjusting for confounding factors, hospitalization due to COVID-19 was associated with an increased risk of HF (hazard ratio = 1.45, 95% confidence interval: 1.39–1.51). Age and race, specifically young age and white race, were identified as risk factors for HF after COVID-19 [128]. Furthermore, it has been demonstrated that acute decompensated HF in the context of a COVID-19 infection was associated with a higher mortality rate than acute decompensated HF without a COVID-19 infection [129]. Further analyses of COVID-19 patients in comparison to the control group revealed an elevated incidence of cardiovascular disease, including arrhythmia, ischemic and non-ischemic cardiomyopathy, as well as HF [130,131]. In the US, mortality rates due to HF increased in the early phases of the pandemic [132]. These data indicate that HF is a significant long-term effect of COVID-19 and may be prevented by adequate primary prevention measures against SARS-CoV-2 infection, as well as close follow-up care for COVID-19 patients [133]. It is noteworthy that the incidence of heart transplantation in 2020 exhibited a decline in many countries, although this trend was not universal [134]. However, there is still a lack of data regarding long-term epidemiology as well as treatment recommendations.

## 9. Current Evidence and Future Trends

In our international HOPE-COVID-19-registry, we conducted multiple subanalyses related to COVID-19 infection. However, the registry, similar to other registries, did not specifically examine COVID-19-associated acute myocarditis [135,136,137,138]. As evidenced by data from the Global Burden of Diseases, Injuries, and Risk Factors Study (GBD), the age-adjusted prevalence of myocarditis remained stable during the initial two years of the pandemic, when compared to the year prior to its onset [139]. The age-adjusted years lived with disability (YLDs) also remained unchanged. However, age-standardized mortality associated with myocarditis has been observed to decline over recent years [139]. One study indicated that at least half of the cases of myocarditis in the initial year of the pandemic were infected with SARS-CoV-2 [140]. In this context, it can be postulated that the prevalence of myocarditis has remained consistent, although there has been an increased prevalence of COVID-19-associated myocarditis. It remains to be seen how the trend will develop over the next few years. In addition, further investigation is necessary to understand the mechanisms and impact of acute myocarditis in COVID-19 patients on short-term as well as long-term follow-up. Similarly, close clinical as well as imaging follow-up should be undertaken to evaluate the vaccination associated myocarditis and SCD at long-term follow-up. Based on these data, treatment algorithms should be evaluated and underpinned by evidence.

## Figures and Tables

**Table 1:** Prevalence of COVID-19-associated acute myocarditis.

	COVID-19-Associated Acute Myocarditis			
Country	Time Period	Sex Male	Prevalence	References
US	March 2020–December 2020	60.4%	2.3% *	[38]
US	January 2021–June 2020	43.5%	5% ^Ω^	[39]
US and Europe	February 2020–April 2021	61.1%	2.4–4.1 per 1000 h.^Ω^	[40]
International	until September 2020	62%	2% ^π^	[42]

* Myocarditis prevalence: 2.3% in COVID-19-positive screened athletes; symptomatic cases: 0.31%. ^Ω^ Myocarditis prevalence in hospitalized patients. ^π^ Myocarditis prevalence in autopsy studies. Abbreviation: US, United States; h, hospitalization.

**Table 2:** Clinical presentation, ECG, troponin, and radiographic findings in COVID-19-associated acute myocarditis.

Clinical Presentation	Prevalence	References
Chest pain	55.5–60%	[40,74,75,76]
Syncope	6%	[40]
Palpitations	11.1%	[40]
Dyspnea	53.7–80%	[40,74,75,76]
Cough	39–67%	[40,75,76]
Fever	57–82.4%	[77,78]
Myalgia	12%	[76]
Gastrointestinal symptoms	33–44.4%	[40,75]
Asymptomatic	1.5–5%	[63,79]
**ECG**		
ST-segment elevation	25.9–28%	[40,75]
Other abnormal ST changes	13–24%	[40,76]
QTc prolongation	25%	[80]
**Laboratory parameters**		
Troponin	86–90%	[75,78]
NT-proBNP	50–87%	[75,78]
**Radiographic findings**		
Cardiomegaly in x-ray	31%	[76]
Bilateral infiltrates in x-ray	31%	[76]
Ground-glass opacities in CT	50%	[76]
**Echocardiographic findings**		
LV dysfunction	20%	[76]
Wall motion abnormalities	20%	[76]
Pericardial effusion	46–67%	[40]
**cMRI**		
T2 elevation	100%	[38]
Late gadolinium enhancement	40.7–50%	[38,76]
**EMB**		
Positive results for myocarditis	82.3%	[40]

Abbreviation: ECG, electrocardiogram; ST-segment, ST segment is the flat, isoelectric section of the ECG between the end of the S wave (the J point) and the beginning of the T wave. QTc, heart-rate corrected QT interval; NT-proBNP, N-terminal pro-brain natriuretic peptide; CT, computer tomography; LV, left ventricular; cMRI, cardiac magnet resonance imaging; EMB, endomyocardial biopsy.
